# Treatment of D-galactose-induced rat polycystic ovarian condition using *Lepidium sativum* and secondary antibodies

**DOI:** 10.5455/javar.2024.k791

**Published:** 2024-06-10

**Authors:** Yousef Alharbi, Abdulrahman Aba Alkhail, Abdel-Kader Zaki

**Affiliations:** 1 Department of Medical Biosciences, College of Veterinary Medicine, Qassim University, Buraydah, Saudi Arabia; 2 National Center for the Prevention & Control of Plants Pests & Animal Diseases, Buraydah, Saudi Arabia; 3 Department of Physiology, Faculty of Veterinary Medicine, Cairo University, Giza, Egypt

**Keywords:** Anti-ovarian antibody, galactose, gonadotrophins, *Lepidium sativum*, PCOS

## Abstract

**Objective:**

There is still much to be discovered regarding the etiopathogenesis and management of polycystic ovarian syndrome (PCOS).

**Materials and Methods:**

Four groups of female Wister-Albino rats were established, each with a normal estrous cycle: control, D ( + ) galactose (D-galactose), *Lepidium sativum* (*L. sativum*), and prepared secondary antibody (Ab2). Serum samples were collected, and histopathological examination was performed on ovaries and spleen tissues. Immunoreactive anti-ovarian antibody (AOA) quantities were determined using a modified antigen-based ELISA procedure. ELISA assay kits were used to quantify FSH, LH, and estradiol 17 β concentrations.

**Results:**

The study found that AOA concentration in undiluted samples was significantly higher in the second and fourth weeks after PCOS induction by D-galactose (*p* < 0.001). However, antibody index% and titer elevated in the D-galactose group. *L. sativum*’s late efficacy was observed in the fourth week, while the concentration of undiluted samples in the D-galactose + Ab2 group lowered (*p* < 0.001). Higher basal FSH and LH levels and lower estrogen levels are associated with PCOS development. *L. sativum*’s immunomodulatory properties may contribute to this association. Estradiol-17ß concentrations increased in D-galactose + *L. sativum* and D-galactose + Ab2 groups, respectively.

**Conclusion:**

Careful extrapolation of experimental models is crucial for clinical applications, as technical advancements make Ab2 production easier. Further study is needed to fully understand its potential in immunotherapy.

## Introduction

Polycystic ovarian syndrome (PCOS), is one of the most common endocrinopathies that cause subfertility, prolonged ovulation failure, and atypical ovarian morphology. According to Daghestani [1] and Ainehchi et al. [2], the etiology and pathogenesis of PCOS are thought to be genetic factors: high levels of LH, glycolipid metabolism disorder, hyperinsulinemia, insulin resistance, hyperandrogenemia, a low-degree inflammatory response, obesity, abnormal ovarian function, and hyper-puberty. Because PCOS includes endocrine, metabolic, and reproductive problems, it can be used as a clinical model for understanding how these components interact [3]. As of this writing, the causes and pathogenesis of PCOS are still not completely known. It is believed to involve abnormalities in steroidogenesis, follicular arrest, a lack of aromatase enzymes, and other factors [4,5]. Over the past 60 years, a large number of animal models have been created and investigated. However, no PCOS animal model has been able to faithfully reproduce the condition’s metabolic and reproductive characteristics. To put it another way, certain animal models are better than others at recreating the metabolic phenotype over the reproductive phenotype [6].

The female rats in the PCOS group had thicker cystic follicle membranes, heavier ovaries, irregular estrous cycles, thicker endometrium, and higher serum concentrations of testosterone, estrogen, LH, and LH/FSH [7,8]. The monosaccharide galactose (D-galactose), which provides energy and galactosylates complex molecules, is crucial for human metabolism [9]. The well-documented detrimental effect of D-galactose on the ovary. D-galactose and its metabolites can cause cystic ovaries, poor follicle numbers, and signs of follicular atresia [10]. They can also impede oocyte growth and decrease the bioactivity of FSH. D-galactose metabolism is a disease that is greatly enriched in PCOS, which is relevant to the relationship between D-galactose and PCOS [11]. The pathogenic mechanisms in premature ovarian failure include the generation of anti-ovarian antibodies (AOA) against ovarian antigens, infiltration of inflammatory cells, elevated levels of proinflammatory cytokines, and decreased Tregs [12]. As a conclusion obtained by Johnson and Laloraya [13], additional research into the altered immunological condition associated with PCOS is necessary. Several ovarian antigens are the target of the AOA class of autoantibodies. A few of the causes of AOA development include laparoscopic abdominal surgery, chronic ovarian inflammation, and oocyte harvesting for use in assisted reproductive techniques [14].

There may be unavoidable negative effects with PCOS medication and surgical treatments [15]. Treatment options include calorie restriction, weight loss, diet and exercise adjustments, and medications including metformin and oral contraceptives [16]. The use of natural treatments for PCOS has drawn more and more attention in recent years. *Lepidium sativum* (*L. sativum*), sometimes referred to as coriander or garden cress, has been proven to have antioxidant, anti-infertility, and anti-inflammatory activities [17]. It is a *Brassicaceae* family annual herb that develops quickly and can grow as tall as 50 cm [18]. *L. sativum*’s secondary metabolites have been found to have estrogenic activity, suggesting that they may be useful in the treatment of PCOS. Sinapic acid and sinapine, which control the metabolism of sex hormones and operate on the male reproductive system to substantially potentiate the hypothalamic-pituitary-gonadal axis’s activity and enhance the reproductive parameters [19], diabetes [18], and cancer [20], among other medical ailments, are all treated with *L. sativum* extract in many different nations.

Secondary antibodies (Ab2) against initial antibodies (Ab1) created for the used antigen have been utilized in the management of autoimmune diseases, in addition to their application in vaccine creation and SARS-CoV-2 infection [21]. Autoantibodies, or antibodies that recognize and target the body’s own tissues, can be prevented from functioning by Ab2 antibodies [22]. The autoantibody’s action can be blocked and tissue damage avoided by using Ab2 antibodies that identify the autoantibody’s antigen-binding region. Here, Ab2 was employed to block the AOA in the hopes of lowering disease activity and enhancing clinical results. The relationship between an autoimmune cause and a cyst was looked into in current research, evaluating the two potential therapeutic choices.

## Materials and Methods

### Ethical approval

All study techniques have been approved by the Committee of Qassim University (No. 23-24-2). Diethyl ether was used as the anesthetic in all surgeries, and every effort was made to lessen the suffering.

### Preparation of rat ovarian tissue protein, Ab1, and Ab2 antibodies

The ovaries of four mature albino rats were removed. After being promptly rinsed in phosphate buffer saline (pH 7.4), the ovaries underwent macroscopical examination, with a focus on the size of the ovarian follicles. The follicular fluid cells, corpora lutea, granulosa, and thecal tissues were homogenized in a solution composed of 0.05 mol/l TRIS-HCL, 0.25 mol/l sucrose, and 1 mmol/l EDTA, pH 7.4 (Staufen, Germany) solution at 0°C–4°C. It was mixed with a cold extraction buffer to homogenize the tissue. After filtering the resulting homogenate through two layers of gauze to remove any remaining tissue fragments, it was centrifuged at 3,000 rpm for 30 min. The upper layer was sonicated on ice for 30 sec at 10 kHz. The protein quantification process was carried out using a colorimetric approach (Biuret reagent) after the sonicates were centrifuged at 10,000 rpm for 30 min at 4°C.

The liquid of the upper layer was used right away as the immunizing antigen. The technique of Chiu and Chamley [23] was used to create the hyperimmune serum against recognized supernatant proteins. The ovarian antigen was administered in the manner described to 4– and 5–month-old male rabbits. First, at least 6–7 subcutaneous sites received 2.0 ml of an emulsion containing an equivalent proportion of produced protein and Freund’s complete adjuvant (Difco, Inc., 0639-60-6, com. 0638-60-7). To optimize a proper negative control for further testing, the first sample was collected before the start of the immunization. The homogenate, which included 400 mg of total proteins, was given to each animal in an amount of 0.5 ml. Following three further injections spaced by 10 days, a final booster dose without the adjuvant was given after 7 days. Three days after the last injection before slaughter, each rabbit provided up to 100 ml of blood, which was then allowed to coagulate. The isolated serum was then kept frozen in aliquots at −20°C.

We precipitated, cleaned, concentrated, and measured the protein content of rabbit serum samples. This combined hyperimmune serum was utilized in the ELISA experiment as a positive control, to make Ab2, and to titrate the strength of the antibodies. Immunoglobulin was precipitated using a 50% saturated ammonium sulfate solution, as reported by Abd El Hafez et al. [24]. After centrifuging the mixture at 4,500 rpm for 20 min, the upper layer was discarded. The precipitate was then disintegrated in PBS, pH 7.2, to adjust the amount of the initial serum sample. Dialysis was used to remove pollutants with small molecular sizes. The produced immunoglobulin precipitate was prepared in compliance with the guidelines provided by the manufacturer, Sigma-Aldrich, and put into a dialysis bag. For 3 days at 4°C, dialysis against 15 mM PBS was performed, with the PBS being changed daily to eliminate the ammonium sulfate. For 20 min, the contents of the bag were centrifuged at 4,500 rpm. The precipitate was extracted from the supernatant, divided into the necessary amounts, and kept in tubes at −20°C until required. Polyethylene glycol produced the concentrated immunoglobulin solution by reducing its water content. Three rabbits of the same breed were immunized to prepare Ab2. Rabbit Ab1 was given subcutaneously at a dose of 40 mg/kg body weight after being thoroughly combined with an equal volume of complete Freund’s adjuvant. The injection schedule remained the same. Two weeks after the original injection, the same inoculation technique was carried out once more. At 21 and 28 days after the first inoculation, booster doses were given without the adjuvant. Blood samples (100 ml) were taken 3 days following the final injection, and the serum was afterward extracted from the blood. The serum was then kept at −20°C until it was required. As previously mentioned, dialyzed and concentrated rabbit serum samples were used to assess the amount of protein contained.

### Checkerboard titration

The maximum concentration, which produced a strong positive signal, was used to achieve the best dilutions of both conjugates. For conjugates of both anti-rabbit and anti-rat IgG in sheep, the optimal dilutions were 1:2000 and 1:1000, respectively. Checkerboard titration of Ab1 and rat ovarian protein antigen was used to determine the ideal antigen dilution and to identify the potency of the prepared hyperimmune sera. A negative control serum and different dilutions of the tested Ab1 were added after the first coating of two ELISA plates (Nunc, Roskilde, Denmark) with a serial dilution of the tested ovarian protein diluted with carbonate bicarbonate buffer pH 9.6 prepared from sodium carbonate (0.159%) and sodium bicarbonate (0.293%). The most ideal condition was found to be the serum dilution with the lowest ovarian protein concentration/well that generated a discernible result; this would be used in subsequent experiments with a known conjugate and substrate dilution. When the potency of the first antibody (Ab1) against the 300 ng ovarian protein was measured, the optimal dilution was 1:16. The produced rat ovarian protein antisera’s potency in the rabbit’s curve controls the amount of antigen needed. The antigen was added to the plate as the negative control after sex point dilutions of ovarian protein, ranging from 250 ng to 15.62 ng (one dilution per row). The plate was washed three times with the washing buffer (PBS pH 7.2 and 0.05% 20), loaded with 100 µl/well of Ab1 (1:16 in PBS), and then incubated for an hour at 37°C while being shaken. After incubation, the plate was incubated for a further hour at 37°C in a shaking water bath with a volume of 100 µl/well of sheep anti-rabbit IgG conjugates (1:2000 dilution in PBS, pH 7.2) added. Following incubation, the plate was rinsed three times before each well received 100 µl of the TMB as substrate solution ([(3,3’,5,5’-tetramethylbenzidine) 30 mg in 75 ml Dw (Sigma Chemical Co.). After allowing the plate to sit at room temperature for 7 min in the dark, the response coloring was visible. To halt the reaction, 50 µl/well of the stopping solution (2.5 M H2SO4) was added. An ELISA reader (BIO-TEK, INC., ELx, 800 UV Winooski, VT, USA) was used to measure the enzyme-mediated reaction at wavelength 492 nm. The relationship between optical density (OD) and antigen log dosage was then displayed on a standard curve. The protocols for the checkerboard titration of Ab2 as antigen were similar, with the exception that ovarian proteins were substituted with consecutive Ab2 five dilutions (5 µg–0.5 µg), which were then followed by the negative control. The OD was then plotted against the log dose of Ab2 as the antigen, and a standard curve was created. The equation that displayed the strength of the prepared rat ovarian protein antibody in rabbits was OD (y) = −0.3112 + 0.3240 log-titer (X), with an *R*^2^ value of 97.8% and an *R*-Sq adj. of 97.3%. The standard log dose-response curve of rat protein antibody was OD (y) = −0.782 + 0.9732 log-conc(X) with an *R*-squared value of 96.5% and an *R*-Sq adj. equal to 94.8%. The potency of the prepared Ab2 was OD (y) = −0.0963 + 0.5215 log-conc(X), with an *R*-squared value of 94.1% and an *R*-squared adj. equal to 91.2%.

### Experimental design

All of the study’s female rats were subjected to vaginal smears. A wet swab containing normal saline was used to perform smears. The vaginal cells were maintained on slides and treated with methylene blue (1%) before being examined under a microscope. The different stages of the rat’s estrous cycle were visible in the experimental groups. Each stage lasts for 1 day. For the D-galactose group, the estrus cycle was irregular and the diestrus phase was extended. Forty normal estrous rats were used in total. They were split into 4 groups, each having 10 rats: rats in the negative control group were fed and watered as usual without receiving any medication injections. According to He et al. [25], the remaining 30 rats received intraperitoneal injections of 200 mg D-galactose/kg/day for 42 days (Riedel De Haen AG Seelze Hannover-Germany, www.lobachemie.com). After the last injection, the estrus cycle was tested for 14 days post-last treatment with D-galactose to ensure an irregular cycle without proestrus and metestrus while the diestrus phase was prolonged [8]. Three groups of rats were formed. D-galactose group: no medication was administered to the rats; they were fed and hydrated normally. D-galactose + *L. sativum *group: the seeds were crushed in an electric mill to a fine powder and stored in an airtight container for subsequent six-time extraction with methanol and concentrated under vacuum using a rotary evaporator before usage at the same dose of D-galactose for 30 days; use of a stomach tube during oral delivery [26]. D-galactose + Ab2 group: The animals received an intraperitoneal injection of an Ab2 produced after its potency was assessed by applying a dosage twice a week for 4 weeks at a rate of 50 mg/kg body weight as an equal time of production of antibodies. To isolate serum, blood samples were taken during the second and fourth weeks following the last day of treatment.

### Measured parameters from ELISA plates

In biological research, ELISA is a diagnostic technique widely used to pinpoint antibodies linked to a specific antigen and distinguish between positive and negative results. Sex readings were taken on each sample. Mean ODs of undiluted samples from the first well of an ELISA plate that were serially diluted. The average undiluted sample’s calibrated concentration. The mean ODs of sample cutoff endpoints from the general formula: cutoff point equal mean ± 3 × standard deviation of the reading of the negative control [27]. The mean calibrated concentration of cutoff endpoints was obtained from the standard dose-response curve. The mean dilution points at which the cutoff endpoints were considered as antibody titers. A positive result is indicated if the dilution point of the tested samples using the newly designed in-house indirect ELISA is greater than the cutoff endpoints, while a negative result is indicated if it is less than the cutoff endpoint. Finally, each sample’s antibody index was computed based on comparisons with standard control values using the formula published by Bauer et al. [28]. The antibody index is equal to (OD of sample—OD of negative)/(OD of positive control—OD of negative)*100.

### Assessment of AOA in rats’ serum 

Regarding Esmailnejad et al. [29], ovarian protein polyclonal antisera (AOA) measurement in rat serum. Each well of the ELISA plate was coated with rat ovarian proteins (300 ng/100 ul carbonate bicarbonate buffer) and incubated for 2 hr at 37°C in a shaking water bath to evenly allocate the protein in the pits. The plates were covered with adhesive tape to prevent evaporation and kept at 4°C overnight to permit the complete adsorption of the solid phase. The three buffer washings removed the extra unattached ovarian proteins. Plates were shaken for 2 hr at 37°C while the remaining empty places were filled with 200 ul/well of bovine serum albumin as a blocking buffer (BSA 2%: pH 7.2). After three rounds of washing, the plates were covered with tape and incubated for 60 min at 37°C while shaken. 100 ul of serially diluted test serum samples were added to each well. All plates were loaded with 100 ul/well of sheep anti-rat IgG conjugates and agitated in a water bath for an hour at 37°C. After three rinsings with washing buffer, a 100 ul/well substrate buffer containing TMB was applied to each well. The plates were incubated for 7 min at room temperature in the dark. The reaction’s unique color was discovered. The response was stopped using a 50 ul/well-stopping solution. The enzyme-mediated response was measured with an ELISA reader at a wavelength of 492 nm.

### Estradiol 17β and gonadotrophin estimation

Manufactured kits for ELISA tests were employed to quantify the various plasma hormone concentrations. Following the manufacturer’s commands, the estradiol 17β Kit (Estradiol ELISA, 17 estradiol antigenic, monocent, lnc. 9025 Eton Ave, Ste C, Canoga Park, CA 91304, USA) was used. The test’s sensitivity was given as 8.7 pg/ml, its intra-assay variability CV as 9.2%, and its inter-assay variability CV as 8.5%. The detection range was 21.23–571.44 pg/ml. Manufactured kits for rat FSH and rat LH were used (Elabscience, USA, Cat. No. E-EL-R0391 and EL-R0026, respectively), and the concentration of FSH and LH were assessed. For FSH and LH, the intra- and inter-assay CVs were 10% and 12%, respectively. The assay ranged from 3.13–200 ng/ml for LH and 0.6–40 ng/ml for FSH, whereas the sensitivity was 1.88 ng/ml and 0.1 ng/ml for LH. The manufacturer’s recommendations were followed when doing each hormone assay in triplicate.

### Macro and microscopical examination

Once the experiment was done, the adult female rats used in the experiment underwent the scarification regimen. The all-rat groups’ postmortem rat female genital system was examined, and any noticeable macroscopical lesions or pathological alterations were noted. It was done using Suvarna et al.’s [30] histological technique. Following examination, ovary and spleen tissue samples from all rats were fixed in 10% formalin.

### Analytical statistics

The means ± standard errors were employed to represent the data values. The data were analyzed using the Kolmogorov-Smirnov test following an ANOVA, where a *p-*value of less than 0.05 was considered statistically significant. The analytical statistics were carried out using SAS program 21 (SAS, USA).

## Results

### AOA, Estradiol 17β, FSH, and LH assay in serum of rat

The outcomes in Table 1 showed that the rabbit hyperimmune sera (Ab1) and preimmunized samples can be read using ELISA settings. The data revealed that there were no significant variations in the reading of the ODs at the cutoff endpoint and calibrated data at the cutoff endpoint between pre- and post-immunization. This resemblance suggests that the cutoff values for the ELISA procedure were chosen correctly. The OD of undiluted samples, calibrated concentration of undiluted samples (ng/100 µl), and dilution point at the cutoff endpoint all demonstrated a statistically significant difference between both before and after vaccination sample findings (*p *< 0.001).

**Table 1.** Approximate ELISA parameters of preimmunized and post-immunized rabbit serum.

Parameters	Preimmunized/post-immunized ^1,2^	Mean ± SE
OD of undiluted samples	1	0.0543 ± 0.0083
	2	1.120 ± 0.0476***
Concentration of undiluted samples (ng/100 µl)	1	1.455 ± 0.1225
	2	248.16 ± 8.432***
OD of cutoff endpoint	1	0.0400 ± 0.0018
	2	0.0410 ± 0.0020
Concentration at cutoff endpoint (ng/100 µl)	1	1.543 ± 0.0742
	2	1.411 ± 0.031
Dilution at the cutoff endpoint	1	2.544 ± 0.1422
	2	248.43 ± 61.423***

Mean ± SE. In the same parameter, values having *** were significantly different from the respective negative control at *p* < 0.001 (ANOVA- Kolmogorov-Smirnov test). 1: Preimmunized. 2: post-immunized.

In this work, the serum of the treated rat groups as well as the normal and D-galactose-induced PCO groups had their levels of antibody against ovarian tissue protein assessed. The approximate ELISA parameters for rat samples from various groups are displayed in Table 2. The findings demonstrated that the groups under investigation had comparable ODs and calibrated concentrations at the cutoff endpoint. This similarity demonstrates that each ELISA raw sample chosen by the study’s cutoff endpoint was appropriate.

According to these findings, in the second (106.723 ± 21.377 *vs.* 3.00 ± 0.577) and fourth weeks (96.041 ± 18.534 *vs.* 2.500 ± 0.500), the dilution at the cutoff endpoint in the D-galactose group was significantly elevated compared to the rat normal sample (*p *< 0.001). The findings showed that at the second and fourth weeks following induction, the concentration of undiluted samples in the D-galactose positive group was significantly higher than that of rat normal samples (96.647 ± 26.922 ng/100l *vs.* 9.3610 ± 2.034 ng/100 µl and 114.722 ± 28.619 *vs.* 13.1301 ± 2.151 ng/100 µl, *p *< 0.01), respectively. However, when compared to the rat normal sample, the antibody index% in the D-galactose group exhibited a significant rise (*p* < 0.001) in the second week (80.766 ± 9.44 *vs.* 7.908 ± 0.130). Like this, after the fourth week, the antibody index% in the D-galactose group considerably elevated (*p *< 0.001) in comparison to the rat normal sample (87.370 ± 9.63 *vs.* 18.392 ± 5.96). In comparison to the D-galactose group, Table 2 displays the estimated ELISA assay parameters for rat samples from the *L. sativum* treated group (D-galactose + *L. sativum*). Based on these findings, it was discovered that the D-galactose + *L. sativum* group’s dilution at the cutoff endpoint significantly decreased (*p *< 0.001) in the fourth week only (10.67 ± 2.67 *vs.* 106.723 ± 21.377). Additionally, only in the fourth week, the antibody index% in the D-galactose + *L. sativum* group was significantly lower (*p* < 0.001) than that in the D-galactose group (48.34 ± 9.28 *vs.* 87.370 ± 9.631).

**Table 2.** Approximate ELISA parameters of sera of rat groups.

Groups ELISA	Control rats		D-galactose		D-gal *+ L. sativum*		D-gal + Ab2		*p-*values
	Wk2	Wk4	Wk2	Wk4	Wk2	Wk4	Wk2	Wk4	
OD of undiluted samples	0.1365 ± 0.0015	0.2618 ± 0.0712	1.2070 ± 0.0994	1.273 ± 0.0963	0.9800 ± 0.017	0.6211 ± 0.111	0.5801 ± 0.069	0.6151 ± 0.147	< 0.001
Concentration of undiluted samples (ng/100 µl)	9.3610 ± 2.034	13.1301 ± 2.151	96.647** ± 26.922	114.722** ± 28.619	66.214 ± 7.419a	28.332 ± 6.68c	24.780 ± 4.42c	28.11 ± 2.18c	< 0.001
OD of cutoff endpoint	0.0445 ± 0.0048	0.0502 ± 0.0074	0.0315 ± 0.0037	0.0303 ± 0.0053	0.0465 ± 0.008	0.0536 ± 0.008	0.0845 ± 0.008	0.0425 ± 0.0037	< 0.2095
Concentration at cutoff endpoint (ng/100 µl)	7.5313 ± 0.086	3.740 ± 2.12	7.303 ± 0.0650	7.2825 ± 0.0923	8.533 ± 0.395	7.698 ± 0.150	7.728 ± 0.484	7.495 ± 0.0664	< 0.175
Dilution at the cutoff endpoint	2.500 ± 0.500	3.000 ± 0.577	96.041 ± 18.534***	106.723 ± 21.377***	87.001 ± 14.577	10.670 ± 2.67c	14.002 ± 1.15c	7.002 ± 1.77c	< 0.001
Antibody index %	7.908 ± 0.130	18.392 ± 5.961	80.766 ± 9.446***	87.370 ± 9.631***	76.79 ± 1.31	48.34 ± 9.28c	44.89 ± 5.78c	18.05 ± 4.87c	< 0.001

Means ± SE. Values with *,**, and *** are significantly different than the control negative group at *p *< 0.05, *p *< 0.01 and *p *< 0.001, respectively. Values with a, b, and c are significantly different than the control positive group at *p *< 0.05, *p *< 0.01, and *p *< 0.001, respectively.

In the D-galactose + *L. sativum group*, the anti-ovarian antibody concentration in undiluted samples was 66.214 ± 7.419 ng/100 µl and 28.33 ± 6.68 ng/100 µl at the second- and fourth-week post-treatment, respectively. The findings also demonstrated that, in the second week following treatment, the concentration of undiluted samples in the D-galactose + *L. sativum* group significantly decreased (*p *< 0.05) compared to that of the D-galactose group (66.214 ± 7.419 ng/100 µl *vs.* 96.647 ± 26.922 ng/100 µl). Additionally, in the fourth week after treatment, the concentration of undiluted samples in the D-galactose + *L. sativum *group was significantly lower (*p* < 0.001) than that of the D-galactose group (28.332 ± 6.68 ng/100 µl *vs.* 114.722 ± 28.619 ng/100 µl).

The results demonstrated that when related to the D-galactose group, the D-galactose + Ab2 group’s dilution at the cutoff endpoint considerably (*p *< 0.001) decreased at the second- and fourth weeks following treatment (14.002 ± 1.15 and 7.002 ± 1.77 *vs.* 96.041 ± 18.534 and 106.723 ± 21.377, respectively). Similar to this, at the second and fourth weeks following treatment with Ab2, the antibody index% in the D-galactose + Ab2 group showed a significant drop compared to that in the D-galactose group (*p *< 0.001) (44.89 ± 5.78 and 18.05 ± 4.87 *vs.* 80.766 ± 9.446 and 87.370 ± 9.631, respectively). The results also showed that at the second and fourth weeks after treatment with Ab2, the concentration of undiluted samples in the D-galactose + Ab2 group significantly decreased (*p *< 0.001) in comparison to that of the D-galactose group (24.780 ± 4.42 and 28.11 ± 2.18 ng/100 µl *vs.* 96.647 ± 26.922 and 114.722 ± 28.619ng/100 µl, respectively).

According to Table 3’s findings, D-galactose rats had serum levels of estradiol 17β that were significantly lower (*p* < 0.001) than those of normal rats (45.118 ± 4.96 pg/ml *vs.* 184.3119 ± 37.43 pg/ml). The findings revealed a significant increase (*p *< 0.001) in the level of estradiol 17β in the serum of the D-galactose + *L. sativum* group as compared to that of the D-galactose group (114.3222 ± 6.46 pg/ml). In comparison to the D-galactose group, the estradiol 17β content in the serum of the D-galactose + Ab2 group was significantly higher (85.431 ± 17.75 pg/ml; *p *< 0.01). FSH levels in the serum of the D-galactose group were significantly higher (12.677 ± 2.285 ng/ml *vs.* 5.025 ± 1.17 ng/ml) than those in the normal rat group (*p* < 0.01). The results revealed a significantly lower level of FSH (6.392 ± 0.716 ng/ml) in the serum of the D-galactose + *L. sativum* group compared to that of the D-galactose group (*p *< 0.01). Additionally, when compared to the D-galactose group, the FSH concentration in the blood of the D-galactose + Ab2 group was significantly lower (4.89 ± 1.00 ng/ml; *p *< 0.05). In terms of LH, the findings revealed that the normal rat group’s serum LH concentration was significantly lower (*p *< 0.05) than that of the D-galactose group’s (10.654 ± 0.75 ng/ml *vs.* 7.435 ± 1.32 ng/ml). In comparison to the D-galactose group, the results demonstrated a significantly lower level of LH in the serum of the D-galactose + *L. sativum* group (7.430 ± 1.03 ng/ml) and the D-galactose + Ab2 group (8.976 ± 0.87 ng/ml).

### Histopathology

**Figure 1.**
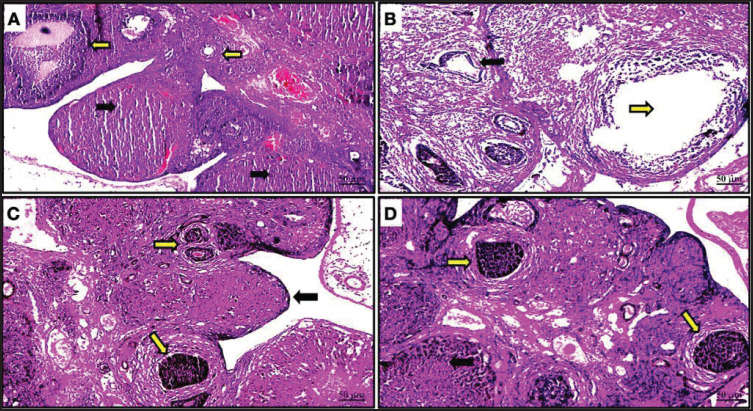
(A) Ovary of the negative control group rats displayed normal well-developed multiple corpora lutea (black arrows) and normal mature follicles in different stages (yellow arrows). Also, moderate congestion in the ovarian blood vessels was noticed. H&E X 40. (B) The ovary of the D-galactose-treated rats showed medium-sized follicular cysts (yellow arrow). On the other spaces degeneration, atrophy, and some mature follicles (black arrow). H&E X 40. (C) The ovary of the Galactose-Garden cress-treated rats showed normal multiple secondary follicles (yellow arrows) and normal well-developed corpora lutea (black arrow). H&E X 40. (D) The ovarian tissue that was treated with both galactose + Ab2 displayed complete recovery from the condition of the ovarian cysts. In association with that; a normal well-developed corpora lutea with also the presence of normal healthy secondary follicles.

**Figure 2.**
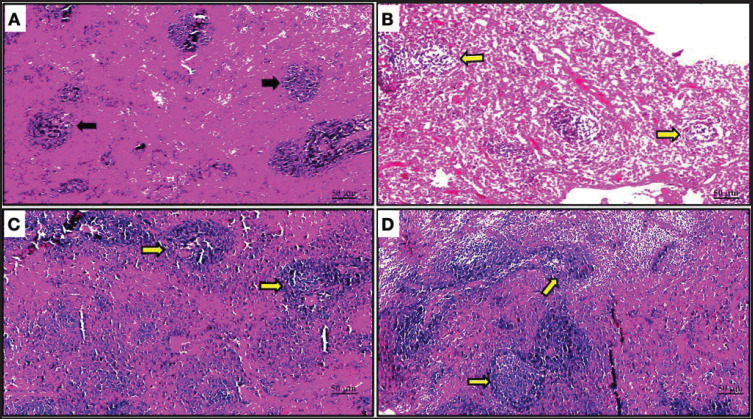
(A) Spleen of the negative control group rats showed normal well-developed lymphoid follicles in the white pulp with the normal splenic artery (black arrows). H&E X 40. (B) Spleen of the D-galactose-treated rats displayed severe lymphoid depletion in most of the lymphoid follicles of the white pulp (yellow arrows) H&E X 40. (C) Spleen of the L Arginine-Glutamine-treated rats showed mild lymphoid depletion in the lymphoid follicles of the white pulp with the normal splenic artery (black arrows). H&E X 40. (D) Spleen of the D-galactose + Ab2-treated rats displayed normal impacted lymphoid follicles in the white pulp and normal splenic artery (yellow arrows) H&E X 40.

The negative control group’s studied ovarian tissue displayed histologically normal architecture and tissue specifics, including numerous well-developed corpora lutea that were present in conjunction with normal ovarian follicles in various stages. Additionally, there was a slight obstruction of the ovarian blood vessels. The white pulp of the spleen contains normal, fully grown lymphoid follicles, and the splenic artery is in good condition. As a positive control group, rats treated with D-galactose to create PCOS in the ovary displayed modest follicular cysts on the ovarian surface. Numerous atretic follicles were seen on the opposite line. The lymphoid follicles in the splenic tissue had modest lymphoid depletion. The ovarian tissue that was treated with both D-galactose and *L. sativum* showed that the polycystic ovarian disease was successfully treated since the ovarian cysts vanished. Additionally, numerous ovarian follicles in various stages as well as healthy, fully developed corpora lutea were found. Normal impacted lymphoid follicles and a normal splenic artery were visible in the splenic tissue. The ovarian tissue that was treated with both D-galactose and Ab2 showed full recovery from the ovarian cyst condition. Additionally, there was a normal, well-developed corpora lutea and a typical, healthy secondary follicle present (Figure 1). The normal impacted lymphoid follicles in the white pulp and the normal splenic arteries were present in the spleen of the D-galactose + Ab2- treated rats (Figs. 1 and 2).

**Table 3.** Concentration of estradiol 17β; FSH and LH of serum of rat with PCOS.

Groups	Parameters		
	Estradio l17β (pg/ml)	FSH (ng/ml)	LH (ng/ml)
Normal	184.311 ± 37.43	5.025 ± 1.17	7.435 ± 1.32
D-galactose	45.118 ± 4.96***	12.677 ± 2.28**	10.654 ± 0.75*
D-galactose + *L. sativum*	114.322 ± 6.46c	6.392 ± 0.716b	7.430 ± 1.03a
D-galactose + Ab2	85.431 ± 17.75b	4.895 ± 1.00c	8.976 ± 0.87a

Means ± SE. Values with *,**, and *** are significantly different than the control normal group at *p *< 0.05, *p *< 0.01, and *p *< 0.001, respectively. Values with a, b, and c are significantly different than the control positive group at *p *< 0.05, *p *< 0.01, and *p *< 0.001, respectively.

## Discussion

The etiology and pathogenesis of PCOS are still not completely known. In our current work, we propose that immunopathology and endocrine system dysfunction add to the complexity of PCOS. A rat model of chronic D-galactose injection was created to validate this problem. Since it provides energy and galactosylates complex molecules, the monosaccharide D-galactose is crucial for both human and animal metabolism [9]. D-galactose performs as a sugar that lowers and interacts with amino groups in biomolecules [31]. The immune system is weakened by D-galactose because of a lack of sex hormones, an increase in reactive oxygen species (ROS), decreased antioxidant enzyme activity, and inflammatory cytokine levels [32].

The well-documented detrimental effect of D-galactose on the ovary. Galactosemia frequently manifests as low follicle numbers and follicular atresia symptoms. Although the exact mechanism by which D-galactose produces these toxic effects on ovaries is unknown, it is known that D-galactose and its end products can impair oocyte growth, decrease the bioactivity of FSH, cause ovarian mortality, and weaken the ovarian response to gonadotropin stimulation. Despite substantial variations in the reproductive physiology of large animals and rodents, studies employing animal models are crucial to investigating the pathophysiology of PCOS *in vivo* [6]. However, no PCOS animal model has been able to faithfully reproduce the condition’s metabolic and reproductive characteristics. Because PCOS is a systemic illness, these animal models are useful for evaluating the pathogenesis of PCOS in diverse tissues. To identify the diagnostic authenticity of AOA in PCOS, the most sensitive and exact cutoff for this test will be looked [33].

AOA is a collection of autoantibodies that attack the lutein follicular cells, theca interna, and the granulosa in the ovary. Plasma, as opposed to oocytes, is also included. The collection of oocytes, laparoscopic abdominal surgery, and chronic ovarian inflammation are only a few causes that can result in AOA. The presence of AOA, whether directed towards ovarian or oocyte tissues, suggests the identification of an autoimmune process that could be the primary or subsequent reaction to an ovarian combination. Endometriosis and other reproductive issues, like adrenal insufficiency, were also linked to AOA [34]. In the present work, the AOA was assessed in the treated rat groups as well as the normal and D-galactose-induced PCOS groups. The findings demonstrated that in the second and fourth weeks, the dilution at the cutoff endpoint and, compared to the rat normal sample, the antibody index% in the D-galactose group were significantly greater. The results showed that at the second and fourth weeks after induction, the calibrated concentration of undiluted samples in the D-galactose group was meaningfully higher than that of rat normal samples. According to a different bioinformatics study, PCOS has a significantly enriched D-galactose metabolism pathology [11].

The study of Jiao et al. [35] demonstrated that the monosaccharide except the glucose biosynthesis pathway can act as a marker for PCOS. The medicinal and surgical treatments for PCOS may unavoidably have negative effects, according to earlier studies [15]. Treatment options include changing one’s diet and exercise routine, reducing calories, losing weight, and taking medications like metformin and oral contraceptives that contain estrogen and progestin [16]. In the present investigation, *L. sativum* and Ab2 were used as experimental treatments. According to the findings, the antibody titer and antibody index% in the D-galactose + *L. sativum* group considerably decreased only in the fourth week. The findings also demonstrated that at just the fourth week after treatment, the concentration of undiluted samples in the D-galactose + *L. sativum* group significantly decreased compared to that of the D-galactose group. At the second and fourth weeks after treatment, the D-galactose + Ab2 group’s dilution at the cutoff endpoint and antibody index% dramatically decreased compared to that of the D-galactose group, according to the data. The findings also showed that during the second and fourth weeks after treatment, the concentration of undiluted samples in the D-galactose + Ab2 group significantly decreased as compared to that of the D-galactose group. The findings demonstrated that, compared to normal rat serum, the concentrations of FSH and LH were considerably higher, and estradiol 17β was significantly decreased in the D-galactose serum. The data presented here is consistent with studies done on mice by Omidi et al. [31] and Ahangarpour et al. [32].

High levels of LH prevent FSH from working properly, which results in the luteinization of granulosa cells, a cessation of small follicle formation, and PCOS [36]. According to Jiang et al. [37], PCOS is a substandard inflammatory reaction that can harm the ovaries, result in polycystic abnormalities, and cause irregular hormone secretion. The female rats in the PCOS group had bigger cystic follicle membranes, irregular estrous cycles, heavier ovaries, and higher serum levels of testosterone, estrogen, LH, and LH/FSH according to the research of Zhang et al. [8]. Additionally, low progesterone levels that the hypothalamus is unable to control have been connected to hormonal dysfunctions in PCOS, which cause a rise in the pulse frequencies of LH and GnRH [7]. Zhang et al.’s research suggest that excess ROS, elevated advanced glycation end products, and decreased antioxidant activity may be involved in the long-term D-galactose therapy’s effects [8].

For the hypothalamus-pituitary gonadal axis (HPG) to generate follicles in the ovary, it required continuous feedback. However, PCOS can develop if this axis is disturbed. Therefore, increased oxidative stress may be to blame for altered HPG axis functioning. The histological analysis of the ovaries of the control group revealed various stages of mature follicles surrounded by stroma. A histological examination of the PCOS group’s ovaries revealed medium cystic follicles that were fluid-filled sacs and atretic follicles. Fewer corpora lutea and antral ovaries were present in PCOS-affected rats [38]. This tendency was supported by the results of PCOS animal models in rats or mice [39]. Thickened theca cells were connected to hyperandrogenemia and increased LH levels, which led to PCOS, according to Chaudhary et al. [40]. According to Walters et al. [41] and Zhang et al. [8], low levels of FSH and higher androgens can limit follicular growth, leading to an increase in atresia follicles and deficiency in granulosa cells.

In comparison to the D-galactose group, the serum of the D-galactose + L. *sativum* group and the D-galactose + Ab2 group exhibited a noteworthy decrease in the concentration of FSH and LH and a considerable elevation in the concentration of estradiol 17β. The immunomodulatory, in addition to anti-inflammatory, properties of *L. sativum* and its major chemical constituents may be the cause of the current data. Alkaloids, flavonoids, triterpenes, phenol derivatives, acetamide, oleic acid, and tocopherol are some of the substances present in *L. sativum* that give it its characteristics [42]. To prevent or treat conditions like PCOS, the plant and its components may be employed. More research into the altered immunological condition in PCOS is necessary, as proposed by Xu et al. [5] and Johnson and Laloraya [13] regarding the possibility that immune disease could play a significant role in the pathophysiology of PCOS.

The use of Ab2 has developed into a crucial tool in immunology and has improved the development of vaccines, cancer therapies, and treatments for autoimmune diseases. As antibodies need to be produced against the first antibody’s variable region, producing Ab2 might be challenging. The immune system can produce a variety of antibodies in response to an Ab-1, according to several studies. However, three different varieties of Ab2 were created based on the first antibody’s antigenic components. Some of them also bind to other antibodies in their class, in addition to the Ab-1 that triggered them. The Ab2 anti-isotypes, which belong to the same class of antibodies, presumably detect antigenic determinants in the constant regions of the heavy and light chains. Anti-allotypes: these are distinct antibodies that react with each antibody made by the particular individual from whom the Ab-1 was derived, but not always with antibodies made by other individuals. When tested against other antibodies, the Ab-2 only connected to the Ab-1. Therefore, it might be said to recognize the uniqueness of the primary antibody (the only “foreign” component). The term “anti-idiotype” was used to describe the Ab-2 that was produced in response to the idiotype antigenic determinants. On the other hand, an anti-idiotype is defined as an antibody that is limited to binding to the Ab-1 that is activated by a specific antigen.

Anti-idiotype antibodies are those Ab2 antibodies that are produced against Ab1 [43]. These antibodies are expected to primarily target the idiotype component. By neutralizing and preventing the generation of autoantibodies, anti-idiotypic antibodies are crucial for maintaining a healthy idiotypic regulatory network and new treatments for autoimmune illnesses, according to Pan et al. [22]. In their review, Stanova et al. [44] conjectured that Ab2 (anti-idiotypic antibodies) could be able to preferentially outcompete an antigen for adhering to an antibody. These exchanges, sometimes referred to as idiotype-anti-idiotype reactions, can be used to influence the immune system, which may have a substantial impact on the treatment of several autoimmune diseases. Because rabbits, as opposed to other laboratory animals such as rats and mice, may produce a more varied, high-affinity repertoire of antibodies [43], rabbits were used in the current investigation to produce therapeutic Ab2.

## Conclusion

The development of autoantibodies against ovarian antigens is one of the pathogenic processes in POF, and this suggests that the illness might be an autoimmune disorder. More research into the altered immunological condition in PCOS is necessary. It is now clear that comparable, trustworthy data collection will be feasible and will contribute to our understanding. Furthermore, there has not been much success in creating effective treatments; immunotherapy is one interesting avenue for future research. Finally, caution should be exercised when extending results from experimental models to clinical demands. Although it can be difficult to produce Ab2, technological advancements have made it easier to do so. To fully comprehend the potential of Ab2 in immunotherapy and to enhance production and application in clinical settings, additional study is required.

## List of Abbreviations

Ab1, Antibody 1; Ab2, Antibody 2; AOA, Anti-ovarian antibody; HPG, Hypothalamus- pituitary-gonadal axis; *L. Sativum, Lepidium sativum; *PCOS, polycystic ovarian syndrome; ROS, reactive oxygen species.
